# *N*-{2-Methyl-5-[(5-oxo-10,11-dihydro-5*H*-dibenzo[*a*,*d*]cyclo­hepten-2-yl)amino]­phen­yl}benzamide

**DOI:** 10.1107/S1600536810024256

**Published:** 2010-06-30

**Authors:** Angelika Dorn, Dieter Schollmeyer, Stefan A. Laufer

**Affiliations:** a Institute of Pharmacy, Department of Pharmaceutical and Medicinal Chemistry, Eberhard Karls University Tübingen, Auf der Morgenstelle 8, 72076 Tübingen, Germany; b Department of Organic Chemistry, Johannes Gutenberg-University Mainz, Duesbergweg 10-14, 55099 Mainz, Germany

## Abstract

In the title compound, C_29_H_24_N_2_O_2_, the two aromatic rings of the tricyclic unit are oriented at a dihedral angle of 32.27 (8)°. In the crystal N—H⋯O hydrogen bonds link the mol­ecules into chains along the *a* axis. Further N—H⋯·O inter­actions link the chains.

## Related literature

For palladium-catalysed amination reactions of aryl halides with anilines, see: Jensen *et al.* (2004 ▶); Grasa *et al.* (2001 ▶). For p38 MAP kinase inhibitors based on dibenzosuberones, see: Laufer *et al.* (2006 ▶). 
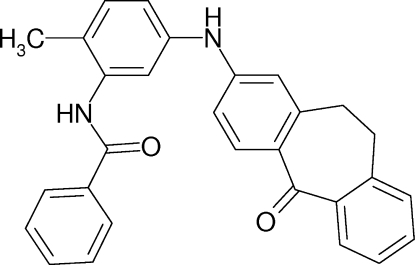


## Experimental

### 

#### Crystal data

C_29_H_24_N_2_O_2_
*M* *_r_* = 432.50
Orthorhombic, 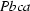

*a* = 8.5878 (6) Å
*b* = 17.0342 (12) Å
*c* = 30.669 (2) Å
*V* = 4486.4 (5) Å^3^
*Z* = 8
Mo *K*α radiation
μ = 0.08 mm^−1^
*T* = 173 K
0.50 × 0.13 × 0.06 mm

#### Data collection

Bruker SMART APEXIIdiffractometer
54075 measured reflections
5388 independent reflections
3740 reflections with *I* > 2σ(*I*)
*R*_int_ = 0.069

#### Refinement

*R*[*F* ^2^ > 2σ(*F* ^2^)] = 0.044
*wR*(*F* ^2^) = 0.119
*S* = 1.00
5388 reflections
301 parameters
H atoms treated by a mixture of independent and constrained refinement
Δρ_max_ = 0.24 e Å^−3^
Δρ_min_ = −0.19 e Å^−3^

### 

Data collection: *APEX2* (Bruker, 2006 ▶); cell refinement: *SAINT* (Bruker, 2006 ▶); data reduction: *SAINT*; program(s) used to solve structure: *SIR97* (Altomare *et al.*, 1999 ▶); program(s) used to refine structure: *SHELXL97* (Sheldrick, 2008 ▶); molecular graphics: *PLATON* (Spek, 2009 ▶); software used to prepare material for publication: *PLATON*.

## Supplementary Material

Crystal structure: contains datablocks I, global. DOI: 10.1107/S1600536810024256/zl2285sup1.cif

Structure factors: contains datablocks I. DOI: 10.1107/S1600536810024256/zl2285Isup2.hkl

Additional supplementary materials:  crystallographic information; 3D view; checkCIF report

## Figures and Tables

**Table 1:** Hydrogen-bond geometry (Å, °)

*D*—H⋯*A*	*D*—H	H⋯*A*	*D*⋯*A*	*D*—H⋯*A*
N17—H17⋯O16^i^	0.88 (2)	2.04 (2)	2.8934 (18)	163 (1)
N25—H25⋯O27^ii^	0.91 (2)	2.01 (2)	2.8566 (17)	156 (1)

Symmetry codes: (i) 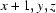; (ii) 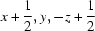.

**Fig. 1.**
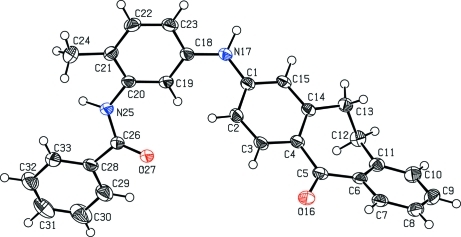
View of compound the title compound. Displacement ellipsoids are drawn at the 50% probability level.

**Fig. 2.**
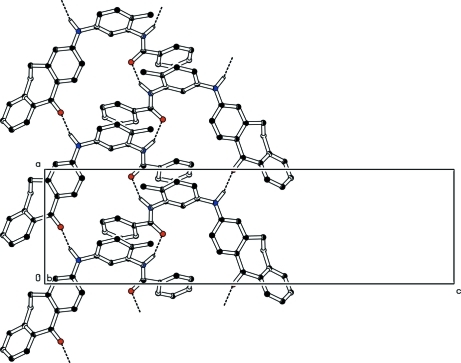
Part of the packing diagram illustrating the hydrogen bonds. View along the b-axis.

## supplementary crystallographic information

### Comment

Recently we designed and synthesized a series of p38 MAP kinase inhibitors based on dibenzosuberones (Laufer *et al.* 2006). The title compound was synthesized in the course of our ongoing studies on dibenzo[*a,d*]cycloheptan-5-ones as potent p38 mitogen-activated protein (MAP) kinase inhibitors.

The structure of the title compound, at 173 (2) K has orthorhombic symmetry. In the molecule (Fig.1), rings A (C1—C4, C14, C15) and B (C6—C11) are, of course, planar and they are oriented at a dihedral angle of A/B = 32.27 (8)°. In the crystal structure N17—H17···O16 (2.89 (2) Å) forms a chain parallel to the a-axis and N25—H25···O27 (2.86 (2) Å) links two by the c-glide plane related chains together (Fig.2).

### Experimental

For the preparation of the title compound a mixture of 500 mg (2.1 mmol) 2-chloro-10,11-dihydro-5*H-*dibenzo[*a,d*]cyclohepten-5-one, 470 mg (2.1 mmol) *N*-(5-amino-2-methylphenyl)benzamide, 940 mg (8.4 mmol) KO*tert-*Bu, 90 mg (0.19 mmol) 2-(dicyclohexylphosphino)-2'-, 4'-, 6'-triisopropylbiphenyl and 20 mg (0.09 mmol) Pd(OAc)_2_ in 3 ml absolute *tert-*butanol and 7 ml absolute toluol was stirred for 3 h at 264 K (90 °C) under an atmosphere of argon. The mixture was diluted with water then extracted with ethyl acetate. The extracts were combined, washed with saturated saline solution, dried over Na_2_SO_4_ and then evaporated under reduced pressure. The residue was purified by flash chromatography (SiO_2_ 60, *n-*hexane / ethyl acetate 7 + 3) (yield: 16.3%). Crystals of the title compound were obtained by slow evaporation of a acetone / ethyl acetate / diethyl ether solution at room temperature. IR (ATR): 3312, 1648, 1575, 1544, 1511, 1292, 1275, 1263, 1109, 763, 701, 689cm^-1^. ^1^H-NMR (DMSO-*d6*) d in ppm: 2.21 (s, 3H), 3.07 (s, 4H), 6.87-7.04 (m, 3H), 7.20-7.59 (m, 8H), 7.84 (d, J=7.8 Hz, 1H), 7.96-8.01 (m, 3H), 8.80 (s, 1H, NH), 9.83 (s, 1H, NH). ^13^C-NMR (DMSO-*d6*) d in ppm: 17.7, 34.4, 36.2, 112.9, 114.3, 118.0, 118.3, 126.8, 127.3, 127.5, 128.0 (x2), 128.8 (x2), 129.0, 130.5, 131.3, 131.9, 132.2, 133.8, 135.0, 137.3, 139.3, 139.4, 142.0, 145.8, 149.0, 165.7, 191.0. HRMS-ESI, *m/z* (C_29_H_24_N_2_O_2_): calcd, 432.1838 [M+H]+; found, 432.1860.

^1^H NMR (200 MHz) and ^13^C NMR (50 MHz) were recorded on a Bruker Advance 200. IR data were determined on a Perkin-Elmer Spectrum One spectrometer (ATR technique). HRMS (EI) (electron impact – high resolution mass spectroscopy) data were obtained from the department for mass spectrometry, Institute of Organic chemistry, Eberhard-Karls-University Tübingen.

### Refinement

Hydrogen atoms attached to carbons were placed at calculated positions with C—H = 0.95 Å (aromatic) or 0.98–0.99 Å (*sp*^3^ C-atom). Hydrogen atoms attached to N17 and N25 were located in diff. Fourier maps and refined using the AFIX 4 constraint (N-H distance is free to refine) with U_iso_(H) = 1.2U_eq_(N). All H atoms attached to carbon atoms were refined in the riding-model approximation with isotropic displacement parameters (set at 1.2–1.5 times of the *U*_eq_(C) of the parent atom).

### Crystal data

**Table:**

C_29_H_24_N_2_O_2_	*F*(000) = 1824
*M**_r_* = 432.50	*D*_x_ = 1.281 Mg m^−^^3^
Orthorhombic, *P**b**c**a*	Mo *K*α radiation, λ = 0.71069 Å
Hall symbol: -P 2ac 2ab	Cell parameters from 6479 reflections
*a* = 8.5878 (6) Å	θ = 2.4–24.1°
*b* = 17.0342 (12) Å	µ = 0.08 mm^−^^1^
*c* = 30.669 (2) Å	*T* = 173 K
*V* = 4486.4 (5) Å^3^	Plate, yellow
*Z* = 8	0.50 × 0.13 × 0.06 mm

### Data collection

**Table:**

Bruker SMART APEXII diffractometer	3740 reflections with *I* > 2σ(*I*)
Radiation source: sealed tube	*R*_int_ = 0.069
graphite	θ_max_ = 27.9°, θ_min_ = 2.4°
CCD scan	*h* = −11→11
54075 measured reflections	*k* = −21→22
5388 independent reflections	*l* = −40→39

### Refinement

**Table:**

Refinement on *F*^2^	Primary atom site location: structure-invariant direct methods
Least-squares matrix: full	Secondary atom site location: difference Fourier map
*R*[*F*^2^ > 2σ(*F*^2^)] = 0.044	Hydrogen site location: inferred from neighbouring sites
*wR*(*F*^2^) = 0.119	H atoms treated by a mixture of independent and constrained refinement
*S* = 1.00	*w* = 1/[σ^2^(*F*_o_^2^) + (0.0522*P*)^2^ + 1.6792*P*] where *P* = (*F*_o_^2^ + 2*F*_c_^2^)/3
5388 reflections	(Δ/σ)_max_ = 0.002
301 parameters	Δρ_max_ = 0.24 e Å^−^^3^
0 restraints	Δρ_min_ = −0.19 e Å^−^^3^

### Special details

**Table:**

Geometry. All esds (except the esd in the dihedral angle between two l.s. planes) are estimated using the full covariance matrix. The cell esds are taken into account individually in the estimation of esds in distances, angles and torsion angles; correlations between esds in cell parameters are only used when they are defined by crystal symmetry. An approximate (isotropic) treatment of cell esds is used for estimating esds involving l.s. planes.
Refinement. Refinement of *F*^2^ against ALL reflections. The weighted *R*-factor *wR* and goodness of fit *S* are based on *F*^2^, conventional *R*-factors *R* are based on *F*, with *F* set to zero for negative *F*^2^. The threshold expression of *F*^2^ > σ(*F*^2^) is used only for calculating *R*-factors(gt) *etc*. and is not relevant to the choice of reflections for refinement. *R*-factors based on *F*^2^ are statistically about twice as large as those based on *F*, and *R*- factors based on ALL data will be even larger.

### Fractional atomic coordinates and isotropic or equivalent isotropic displacement parameters (Å^2^)

**Table:**

	*x*	*y*	*z*	*U*_iso_*/*U*_eq_	
C1	0.56067 (17)	0.43978 (9)	0.43200 (5)	0.0264 (3)	
C2	0.42391 (17)	0.45759 (10)	0.40902 (5)	0.0303 (4)	
H2	0.4294	0.4810	0.3810	0.036*	
C3	0.28200 (17)	0.44081 (10)	0.42747 (5)	0.0294 (3)	
H3	0.1905	0.4539	0.4117	0.035*	
C4	0.26547 (17)	0.40522 (9)	0.46867 (5)	0.0266 (3)	
C5	0.10305 (18)	0.39527 (9)	0.48427 (5)	0.0291 (3)	
C6	0.05457 (17)	0.35895 (9)	0.52689 (5)	0.0278 (3)	
C7	−0.08844 (18)	0.38403 (10)	0.54369 (5)	0.0327 (4)	
H7	−0.1429	0.4252	0.5294	0.039*	
C8	−0.1525 (2)	0.35043 (11)	0.58062 (5)	0.0382 (4)	
H8	−0.2491	0.3689	0.5917	0.046*	
C9	−0.0751 (2)	0.28965 (11)	0.60145 (6)	0.0416 (4)	
H9	−0.1197	0.2652	0.6263	0.050*	
C10	0.0673 (2)	0.26490 (10)	0.58569 (6)	0.0375 (4)	
H10	0.1208	0.2239	0.6004	0.045*	
C11	0.13471 (18)	0.29860 (9)	0.54876 (5)	0.0295 (3)	
C12	0.29383 (19)	0.27248 (9)	0.53467 (6)	0.0332 (4)	
H12A	0.2876	0.2489	0.5052	0.040*	
H12B	0.3329	0.2319	0.5550	0.040*	
C13	0.40667 (18)	0.34182 (10)	0.53394 (5)	0.0308 (4)	
H13A	0.3797	0.3783	0.5579	0.037*	
H13B	0.5136	0.3223	0.5393	0.037*	
C14	0.40373 (17)	0.38601 (9)	0.49132 (5)	0.0249 (3)	
C15	0.54564 (17)	0.40451 (9)	0.47287 (5)	0.0260 (3)	
H15	0.6376	0.3927	0.4888	0.031*	
O16	−0.00360 (13)	0.42043 (8)	0.46106 (4)	0.0426 (3)	
N17	0.70774 (15)	0.45536 (8)	0.41644 (4)	0.0314 (3)	
H17	0.7877 (18)	0.45103 (13)	0.4342 (4)	0.038*	
C18	0.74679 (17)	0.47550 (9)	0.37302 (5)	0.0272 (3)	
C19	0.68395 (17)	0.43432 (9)	0.33809 (5)	0.0266 (3)	
H19	0.6149	0.3917	0.3433	0.032*	
C20	0.72163 (16)	0.45516 (9)	0.29563 (5)	0.0254 (3)	
C21	0.82292 (18)	0.51809 (9)	0.28699 (5)	0.0297 (3)	
C22	0.89126 (19)	0.55434 (10)	0.32282 (6)	0.0350 (4)	
H22	0.9662	0.5944	0.3179	0.042*	
C23	0.85449 (18)	0.53435 (10)	0.36533 (5)	0.0332 (4)	
H23	0.9027	0.5608	0.3890	0.040*	
C24	0.8502 (2)	0.54853 (11)	0.24131 (6)	0.0401 (4)	
H24A	0.9054	0.5988	0.2427	0.060*	
H24B	0.7499	0.5559	0.2266	0.060*	
H24C	0.9130	0.5106	0.2249	0.060*	
N25	0.66532 (14)	0.41082 (8)	0.25969 (4)	0.0283 (3)	
H25	0.7321 (14)	0.3984 (3)	0.2380 (5)	0.034*	
C26	0.53364 (17)	0.36762 (9)	0.25892 (5)	0.0277 (3)	
O27	0.43664 (12)	0.36770 (7)	0.28857 (4)	0.0352 (3)	
C28	0.51003 (18)	0.31897 (10)	0.21871 (5)	0.0301 (4)	
C29	0.4196 (2)	0.25209 (11)	0.22279 (6)	0.0466 (5)	
H29	0.3747	0.2390	0.2501	0.056*	
C30	0.3948 (3)	0.20449 (13)	0.18704 (7)	0.0627 (6)	
H30	0.3353	0.1578	0.1901	0.075*	
C31	0.4558 (3)	0.22431 (13)	0.14716 (7)	0.0577 (6)	
H31	0.4380	0.1913	0.1227	0.069*	
C32	0.5426 (2)	0.29157 (13)	0.14238 (6)	0.0496 (5)	
H32	0.5829	0.3055	0.1146	0.060*	
C33	0.5709 (2)	0.33890 (12)	0.17818 (6)	0.0385 (4)	
H33	0.6321	0.3850	0.1751	0.046*	

### Atomic displacement parameters (Å^2^)

**Table:**

	*U*^11^	*U*^22^	*U*^33^	*U*^12^	*U*^13^	*U*^23^
C1	0.0229 (7)	0.0303 (8)	0.0260 (8)	0.0015 (6)	−0.0023 (6)	−0.0063 (6)
C2	0.0257 (8)	0.0420 (9)	0.0232 (8)	0.0040 (7)	−0.0026 (6)	0.0007 (7)
C3	0.0229 (8)	0.0403 (9)	0.0248 (8)	0.0057 (6)	−0.0060 (6)	−0.0025 (7)
C4	0.0232 (7)	0.0314 (8)	0.0252 (8)	0.0016 (6)	−0.0037 (6)	−0.0038 (6)
C5	0.0239 (8)	0.0335 (8)	0.0300 (8)	0.0000 (6)	−0.0058 (6)	−0.0047 (7)
C6	0.0235 (7)	0.0324 (8)	0.0274 (8)	−0.0059 (6)	−0.0030 (6)	−0.0078 (6)
C7	0.0257 (8)	0.0392 (9)	0.0330 (9)	−0.0041 (7)	−0.0041 (7)	−0.0088 (7)
C8	0.0281 (8)	0.0500 (11)	0.0364 (10)	−0.0085 (8)	0.0030 (7)	−0.0108 (8)
C9	0.0409 (10)	0.0458 (11)	0.0380 (10)	−0.0154 (8)	0.0058 (8)	−0.0030 (8)
C10	0.0412 (10)	0.0314 (9)	0.0397 (10)	−0.0072 (7)	0.0003 (8)	−0.0008 (7)
C11	0.0302 (8)	0.0267 (8)	0.0315 (8)	−0.0049 (6)	−0.0016 (7)	−0.0057 (6)
C12	0.0348 (9)	0.0298 (8)	0.0350 (9)	0.0036 (7)	−0.0006 (7)	0.0012 (7)
C13	0.0258 (8)	0.0375 (9)	0.0292 (8)	0.0037 (7)	−0.0034 (7)	0.0034 (7)
C14	0.0249 (7)	0.0251 (7)	0.0248 (8)	0.0020 (6)	−0.0034 (6)	−0.0048 (6)
C15	0.0224 (7)	0.0307 (8)	0.0249 (8)	0.0038 (6)	−0.0073 (6)	−0.0044 (6)
O16	0.0218 (6)	0.0675 (9)	0.0384 (7)	0.0022 (6)	−0.0066 (5)	0.0083 (6)
N17	0.0207 (6)	0.0500 (9)	0.0235 (7)	0.0001 (6)	−0.0050 (5)	−0.0013 (6)
C18	0.0203 (7)	0.0350 (8)	0.0263 (8)	0.0023 (6)	−0.0020 (6)	−0.0018 (6)
C19	0.0193 (7)	0.0315 (8)	0.0292 (8)	−0.0014 (6)	−0.0010 (6)	−0.0007 (6)
C20	0.0183 (7)	0.0313 (8)	0.0266 (8)	0.0007 (6)	−0.0017 (6)	−0.0035 (6)
C21	0.0250 (8)	0.0324 (8)	0.0317 (8)	−0.0010 (6)	0.0027 (7)	0.0006 (7)
C22	0.0298 (9)	0.0342 (9)	0.0411 (10)	−0.0095 (7)	0.0014 (7)	−0.0031 (7)
C23	0.0272 (8)	0.0388 (9)	0.0336 (9)	−0.0039 (7)	−0.0041 (7)	−0.0094 (7)
C24	0.0409 (10)	0.0405 (10)	0.0390 (10)	−0.0062 (8)	0.0048 (8)	0.0061 (8)
N25	0.0202 (6)	0.0398 (8)	0.0248 (7)	−0.0032 (5)	0.0022 (5)	−0.0048 (6)
C26	0.0211 (7)	0.0364 (9)	0.0255 (8)	−0.0003 (6)	−0.0022 (6)	0.0008 (6)
O27	0.0225 (5)	0.0576 (8)	0.0253 (6)	−0.0072 (5)	0.0001 (5)	−0.0021 (5)
C28	0.0229 (7)	0.0383 (9)	0.0291 (8)	0.0008 (7)	−0.0061 (6)	−0.0029 (7)
C29	0.0565 (12)	0.0457 (11)	0.0377 (10)	−0.0158 (9)	−0.0097 (9)	0.0012 (8)
C30	0.0861 (17)	0.0457 (12)	0.0562 (14)	−0.0244 (12)	−0.0216 (12)	−0.0030 (10)
C31	0.0736 (15)	0.0564 (13)	0.0431 (12)	0.0011 (11)	−0.0194 (11)	−0.0191 (10)
C32	0.0470 (11)	0.0682 (14)	0.0337 (10)	0.0006 (10)	−0.0015 (9)	−0.0120 (9)
C33	0.0311 (9)	0.0524 (11)	0.0320 (9)	−0.0058 (8)	−0.0004 (7)	−0.0061 (8)

### Geometric parameters (Å, °)

**Table:**

C1—N17	1.3760 (19)	N17—H17	0.880 (19)
C1—C15	1.396 (2)	C18—C23	1.384 (2)
C1—C2	1.403 (2)	C18—C19	1.390 (2)
C2—C3	1.374 (2)	C19—C20	1.388 (2)
C2—H2	0.9500	C19—H19	0.9500
C3—C4	1.409 (2)	C20—C21	1.406 (2)
C3—H3	0.9500	C20—N25	1.4210 (19)
C4—C14	1.414 (2)	C21—C22	1.390 (2)
C4—C5	1.484 (2)	C21—C24	1.512 (2)
C5—O16	1.2366 (19)	C22—C23	1.384 (2)
C5—C6	1.505 (2)	C22—H22	0.9500
C6—C7	1.399 (2)	C23—H23	0.9500
C6—C11	1.407 (2)	C24—H24A	0.9800
C7—C8	1.383 (2)	C24—H24B	0.9800
C7—H7	0.9500	C24—H24C	0.9800
C8—C9	1.386 (3)	N25—C26	1.3494 (19)
C8—H8	0.9500	N25—H25	0.905 (18)
C9—C10	1.381 (3)	C26—O27	1.2334 (18)
C9—H9	0.9500	C26—C28	1.499 (2)
C10—C11	1.395 (2)	C28—C29	1.384 (2)
C10—H10	0.9500	C28—C33	1.391 (2)
C11—C12	1.501 (2)	C29—C30	1.380 (3)
C12—C13	1.528 (2)	C29—H29	0.9500
C12—H12A	0.9900	C30—C31	1.373 (3)
C12—H12B	0.9900	C30—H30	0.9500
C13—C14	1.509 (2)	C31—C32	1.374 (3)
C13—H13A	0.9900	C31—H31	0.9500
C13—H13B	0.9900	C32—C33	1.384 (2)
C14—C15	1.380 (2)	C32—H32	0.9500
C15—H15	0.9500	C33—H33	0.9500
N17—C18	1.415 (2)		
			
N17—C1—C15	118.63 (13)	C1—N17—C18	126.19 (13)
N17—C1—C2	123.55 (14)	C1—N17—H17	118.98 (8)
C15—C1—C2	117.82 (14)	C18—N17—H17	114.78 (8)
C3—C2—C1	119.41 (15)	C23—C18—C19	119.58 (14)
C3—C2—H2	120.3	C23—C18—N17	119.62 (14)
C1—C2—H2	120.3	C19—C18—N17	120.73 (14)
C2—C3—C4	123.25 (14)	C20—C19—C18	120.25 (14)
C2—C3—H3	118.4	C20—C19—H19	119.9
C4—C3—H3	118.4	C18—C19—H19	119.9
C3—C4—C14	117.11 (14)	C19—C20—C21	121.08 (14)
C3—C4—C5	115.65 (13)	C19—C20—N25	120.82 (14)
C14—C4—C5	127.18 (14)	C21—C20—N25	118.02 (13)
O16—C5—C4	118.09 (14)	C22—C21—C20	116.81 (14)
O16—C5—C6	115.95 (14)	C22—C21—C24	120.97 (15)
C4—C5—C6	125.93 (13)	C20—C21—C24	122.13 (14)
C7—C6—C11	118.48 (15)	C23—C22—C21	122.61 (15)
C7—C6—C5	115.94 (14)	C23—C22—H22	118.7
C11—C6—C5	125.38 (14)	C21—C22—H22	118.7
C8—C7—C6	121.63 (16)	C22—C23—C18	119.42 (15)
C8—C7—H7	119.2	C22—C23—H23	120.3
C6—C7—H7	119.2	C18—C23—H23	120.3
C7—C8—C9	119.75 (16)	C21—C24—H24A	109.5
C7—C8—H8	120.1	C21—C24—H24B	109.5
C9—C8—H8	120.1	H24A—C24—H24B	109.5
C10—C9—C8	119.40 (17)	C21—C24—H24C	109.5
C10—C9—H9	120.3	H24A—C24—H24C	109.5
C8—C9—H9	120.3	H24B—C24—H24C	109.5
C9—C10—C11	121.72 (17)	C26—N25—C20	126.07 (13)
C9—C10—H10	119.1	C26—N25—H25	113.04 (9)
C11—C10—H10	119.1	C20—N25—H25	118.67 (8)
C10—C11—C6	118.99 (15)	O27—C26—N25	123.53 (14)
C10—C11—C12	119.30 (15)	O27—C26—C28	121.05 (14)
C6—C11—C12	121.65 (14)	N25—C26—C28	115.42 (13)
C11—C12—C13	110.64 (13)	C29—C28—C33	119.51 (16)
C11—C12—H12A	109.5	C29—C28—C26	117.15 (15)
C13—C12—H12A	109.5	C33—C28—C26	123.32 (15)
C11—C12—H12B	109.5	C30—C29—C28	119.88 (19)
C13—C12—H12B	109.5	C30—C29—H29	120.1
H12A—C12—H12B	108.1	C28—C29—H29	120.1
C14—C13—C12	112.82 (13)	C31—C30—C29	120.3 (2)
C14—C13—H13A	109.0	C31—C30—H30	119.9
C12—C13—H13A	109.0	C29—C30—H30	119.9
C14—C13—H13B	109.0	C30—C31—C32	120.45 (18)
C12—C13—H13B	109.0	C30—C31—H31	119.8
H13A—C13—H13B	107.8	C32—C31—H31	119.8
C15—C14—C4	119.15 (14)	C31—C32—C33	119.76 (19)
C15—C14—C13	117.03 (13)	C31—C32—H32	120.1
C4—C14—C13	123.73 (14)	C33—C32—H32	120.1
C14—C15—C1	123.23 (14)	C32—C33—C28	120.05 (17)
C14—C15—H15	118.4	C32—C33—H33	120.0
C1—C15—H15	118.4	C28—C33—H33	120.0
			
N17—C1—C2—C3	−179.11 (15)	N17—C1—C15—C14	−179.60 (14)
C15—C1—C2—C3	0.7 (2)	C2—C1—C15—C14	0.5 (2)
C1—C2—C3—C4	−0.8 (3)	C15—C1—N17—C18	166.23 (15)
C2—C3—C4—C14	−0.4 (2)	C2—C1—N17—C18	−13.9 (3)
C2—C3—C4—C5	177.03 (15)	C1—N17—C18—C23	137.86 (17)
C3—C4—C5—O16	−2.0 (2)	C1—N17—C18—C19	−45.3 (2)
C14—C4—C5—O16	175.13 (15)	C23—C18—C19—C20	−3.9 (2)
C3—C4—C5—C6	179.85 (14)	N17—C18—C19—C20	179.24 (14)
C14—C4—C5—C6	−3.0 (3)	C18—C19—C20—C21	−0.3 (2)
O16—C5—C6—C7	−24.6 (2)	C18—C19—C20—N25	176.39 (14)
C4—C5—C6—C7	153.62 (15)	C19—C20—C21—C22	4.4 (2)
O16—C5—C6—C11	150.24 (16)	N25—C20—C21—C22	−172.33 (14)
C4—C5—C6—C11	−31.6 (2)	C19—C20—C21—C24	−172.18 (15)
C11—C6—C7—C8	−0.7 (2)	N25—C20—C21—C24	11.1 (2)
C5—C6—C7—C8	174.43 (14)	C20—C21—C22—C23	−4.7 (2)
C6—C7—C8—C9	−0.9 (2)	C24—C21—C22—C23	171.97 (16)
C7—C8—C9—C10	1.8 (3)	C21—C22—C23—C18	0.7 (3)
C8—C9—C10—C11	−1.1 (3)	C19—C18—C23—C22	3.7 (2)
C9—C10—C11—C6	−0.5 (2)	N17—C18—C23—C22	−179.41 (15)
C9—C10—C11—C12	176.51 (15)	C19—C20—N25—C26	26.7 (2)
C7—C6—C11—C10	1.4 (2)	C21—C20—N25—C26	−156.54 (15)
C5—C6—C11—C10	−173.26 (14)	C20—N25—C26—O27	7.6 (3)
C7—C6—C11—C12	−175.52 (14)	C20—N25—C26—C28	−173.21 (14)
C5—C6—C11—C12	9.8 (2)	O27—C26—C28—C29	−26.9 (2)
C10—C11—C12—C13	−121.01 (16)	N25—C26—C28—C29	153.85 (16)
C6—C11—C12—C13	55.9 (2)	O27—C26—C28—C33	151.57 (17)
C11—C12—C13—C14	−86.65 (17)	N25—C26—C28—C33	−27.7 (2)
C3—C4—C14—C15	1.6 (2)	C33—C28—C29—C30	2.1 (3)
C5—C4—C14—C15	−175.47 (15)	C26—C28—C29—C30	−179.38 (18)
C3—C4—C14—C13	−174.79 (14)	C28—C29—C30—C31	−1.8 (3)
C5—C4—C14—C13	8.1 (2)	C29—C30—C31—C32	0.2 (4)
C12—C13—C14—C15	−133.61 (14)	C30—C31—C32—C33	1.2 (3)
C12—C13—C14—C4	42.9 (2)	C31—C32—C33—C28	−0.9 (3)
C4—C14—C15—C1	−1.8 (2)	C29—C28—C33—C32	−0.7 (3)
C13—C14—C15—C1	174.91 (14)	C26—C28—C33—C32	−179.17 (16)

### Hydrogen-bond geometry (Å, °)

**Table:**

*D*—H···*A*	*D*—H	H···*A*	*D*···*A*	*D*—H···*A*
N17—H17···O16^i^	0.88 (2)	2.04 (2)	2.8934 (18)	163 (1)
N25—H25···O27^ii^	0.91 (2)	2.01 (2)	2.8566 (17)	156 (1)

Symmetry codes: (i) *x*+1, *y*, *z*; (ii) *x*+1/2, *y*, −*z*+1/2.
